# Pembrolizumab monotherapy survival benefits in metastatic non-small-cell lung cancer: a systematic review of real-world data

**DOI:** 10.1007/s12672-024-01153-3

**Published:** 2024-07-24

**Authors:** Tomasz Macioch, Maciej Krzakowski, Klaudia Gołębiewska, Małgorzata Dobek, Natalia Warchałowska, Maciej Niewada

**Affiliations:** 1 Department of Experimental and Clinical Pharmacology, Medical University of Warsaw, Żwirki i Wigury 81, 02-091 Warsaw, Poland; 2 HealthQuest Sp. z.o.o., Warsaw, Poland; 3 Department of Lung & Thoracic Tumours, The Maria Sklodowska-Curie National Research Institute of Oncology, Warsaw, Poland

**Keywords:** Pembrolizumab, Non-small-cell lung cancer, Overall survivor, Real world evidence, Monotherapy, Performance status

## Abstract

The efficacy of pembrolizumab in the treatment-naïve non-small-cell lung cancer (NSCLC) patients was proved in the KEYNOTE-024 randomized trial. The aim of this systematic literature review was to identify and summarize the real world evidence (RWE) of overall survival (OS) in previously untreated patients with NSCLC receiving pembrolizumab monotherapy. A systematic search was conducted in PubMed (MEDLINE®) and EMBASE databases. Analyses were focused on survival data (median OS and survival rates at specific time points). To explore the population comparable with the KEYNOTE-024 study, we focused on studies enrolling at least 50% of patients at stage IV of cancer and ECOG performance status 0–2. A total of 41 RWE studies covering over 7600 advanced NSCLC patients naïve to systemic treatment were identified. Overall, survival outcomes reported in those studies vary considerably (median OS range: 3.0–34.6 months). Most RWE studies reported median OS shorter to that reported in KEYNOTE-024 (26.3 months), but about half of reported OS medians were in range of 95% confidence interval for OS as reported in KEYNOTE-024 trial (18.3–40.4 months). Patients with similar characteristics of stage and performance status to those of KEYNOTE-024 trial benefited the same with pembrolizumab monotherapy as their survival outcomes (18.9–22.8 months) were consistent with those reported in the clinical trial. RWE data showed substantially worse outcomes in patients with ECOG-PS 2+ compared to ECOG-PS 0–1 patients.

**Supplementary Information:**

The online version contains supplementary material available at 10.1007/s12672-024-01153-3.

## Introduction

Primary lung cancer is the 2nd most common malignancy after breast cancer, and leading cause of deaths due to malignancy worldwide. Lung cancer accounted for approximately 1.8 million deaths in 2020 year [1]. Non-small-cell lung carcinoma (NSCLC) accounts for 80%–90% of lung cancers, and most patients with NSCLC present with advanced-stage unresectable disease (stage IIIB to IV) [2]. Therefore, patients with NSCLC are at great need for effective and safe systemic therapy that can prolong their life and improve its quality. Until recently, lung cancers were considered poorly immunogenic i.e. minimal benefit have been seen in studies of vaccines or cytokine modulation [3]. However, monoclonal antibodies directed against the immune-checkpoint molecules, such as programmed cell death 1 (PD-1) receptor or its ligand (PD-L1) significantly improved NSCLC therapy outcomes. Currently, patients with locally advanced and unresectable or metastatic NSCLC with no activating genetic abnormalities (*EGFR*, *ALK* or *ROS1*) should be offered immunotherapy as monotherapy or combined with chemotherapy as a standard approach [2].

Pembrolizumab is a humanized monoclonal antibody against PD-1 that has increased activity in tumours which express PD-L1. Pembrolizumab is approved as monotherapy for the first-line treatment of metastatic NSCLC in adults with PD-L1 expression on at least 50% of tumour cells with no *EGFR* mutation or *ALK* fusion [4]. Efficacy of pembrolizumab in the first-line treatment of patients with metastatic NSCLC and high PD-L1 expression was assessed in a randomised multicentre, open-label, controlled KEYNOTE-024 study. Patients were randomised (1:1) to receive pembrolizumab at a dose of 200 mg every 3 weeks (n = 154) or investigator’s choice platinum-containing chemotherapy (n = 151). Among the 154 patients treated with pembrolizumab in KEYNOTE-024 (median age 64.5 years), 59.7% were male and 35.1% and 64.9% of patients had ECOG performance status 0 and 1, respectively. The majority of patients (81.2%) had non-squamous-cell carcinoma (squamous-cell carcinoma in 18.8%). Brain metastases were present at 11.7% of patients. At 5 year follow-up (median time from randomization 59.9 months), 103 patients (66.9%) in the pembrolizumab group have died. Median overall survival (OS) was 26.3 months (95% CI 18.3 to 40.4 months) and Kaplan–Meier estimate of OS at 5 years was 31.9% [5]. Therefore, pembrolizumab as monotherapy has shown durable efficacy regarding OS for the first-line treatment of metastatic NSCLC in adults whose tumours express PD-L1 with a ≥ 50% tumour proportion score (TPS) under controlled clinical trial. However, it is also important to analyse the real world effectiveness beyond the strictly controlled environment of clinical trial, since those studies provides data for broader populations and includes patients typically excluded or underrepresented in clinical trials.

## Purpose of the analysis

The aim of this systematic literature review was to identify and summarize the real world evidence (RWE) of OS in previously untreated patients with NSCLC with high PD-L1 status receiving pembrolizumab monotherapy.

## Methods

A systematic review of observational studies on pembrolizumab monotherapy in previously untreated NSCLC patients was performed. Systematic search was conducted in PubMed (MEDLINE®) and EMBASE databases. The search strategy included both Text Words and MeSH terms for NSCLC and pembrolizumab, coupled with queries about actual study designs regarding real world evidence and corresponding synonyms. Search strategies are presented in Tables 1 and 2 (Supplementary materials). The cut-off date was 17th June 2022. No geographic restrictions were imposed, however, the search was limited to studies published in English. Only full-text publications were reviewed, abstracts and other conference reports were excluded.

The studies were selected independently by three researchers (N.W.; K.G.; M.D.). All studies were assessed according to the eligibility criteria:

1. RWE studies—publications with the hallmarks of clinical trials, such as sample size determination, randomization etc., were excluded;
2. use of pembrolizumab as monotherapy in first-line treatment—studies in previously treated patients as well as those in which pembrolizumab was administered as part of a combination therapy were excluded;
3. presence of PD-L1 expression with a tumour proportion score (TPS) ≥ 50%;
4. performance status according to the Eastern Cooperative Oncology Group (ECOG) scale ≤ 2;
5. presence of distant metastases reported explicitly in the characteristics of the population of a given study or determined by the stage of the disease i.e. stage IV (studies in which patients with stage IV represented at least 50% of study population were allowed);
6. presented data for OS or survival rates.

We developed a standardised data extraction form in MS Excel. Key data were extracted from all studies that met the inclusion criteria for the review, including study design, patient characteristics at baseline, and efficacy endpoints (both median OS and survival rates at individual time points). The data extraction was independently verified and validated; any discrepancies between reviewers were resolved through discussion or consultation with a third reviewer if necessary.

Analyses were focused on survival data (median OS and survival rates at specific time points). Although KEYNOTE-024 trial included only stage IV and ECOG-PS 0–1, we decided to include data from studies in which at least 50% patients had IV stage of cancer and ECOG performance status 0–2. Additional explorative analyses covered data from studies which reported separately ECOG-PS 0–1 and ECOG-PS 2 patients. Wherever possible, data for population similar to KEYNOTE-024 trial (i.e. only patients at IV stage of disease and with ECOG-PS 0–1) were extracted. Correlation between median OS and ECOG was explored.

Forest plots were generated to summarize median OS for the overall study populations and subpopulation groups of interest. We did not pool estimates of median OS or survival rates since meta-analysis methods for median survival ratio are not appropriate [6]. Data are summarized with median (range: min.–max.) statistics. For correlation of median OS and ECOG, brain or liver metastasis status or disease stage Spearman’s correlation coefficient were used [7]. We assumed correlation > 0.8 to be very strong, 0.6–0.8 to be moderately strong, 0.3–0.5 to be fair and < 0.3 to be poor [8]. Hazard ratio (HR) for OS data in predefined populations recognized by ECOG, brain or liver metastasis status or cancer stage were pooled with fixed effect inverse variance approach in Review Manager (RevMan), Version 5.4.1, The Cochrane Collaboration, 2020.

## Results

Through search in PubMed and EMBASE, we identified 825 publications and based on title and abstract we selected 112 potential studies (Supplementary materials, Fig. 1). After full-text review, we identified 41 RWE studies covering 46 cohorts of advanced NSCLC patients naïve to systemic treatment (Table 1). Most patients included in those studies had ECOG-PS 0–1 (median 81.6%), were at the IV stage of the disease (median 85.8%). A minority of patients presented squamous-cell carcinoma (median 21.7%). Other types of NSCLC were reported inconsistently. In 13 studies, all populations were at the IV stage of the disease, and in 6 studies, all patients had ESOG 0–1. Only 3 studies [9, 18, 48—EHR cohort] reported OS in populations similar to KEYNOTE-024 i.e. all patients at IV stage of the disease and with ECOG-PS 0–1. Median age of patients treated with pembrolizumab was 69 years. Most patients were male (median = 65.7%), and current or ex-smokers (median = 90.0%). Almost fifth of all patients had brain metastases (median = 19.3%) and 13.0% had liver metastases. Most of patients with brain metastases (median = 68.8%) had undergone previous local therapy (surgery or radiotherapy). Table 2 summarize patients’ characteristics in studies included in this review.

**Table 1:** Characteristics of the studies included in the analysis

Study	Duration time	Number and location of centres	Number of patients	Median age	Males (%)	Current or previous smokers	Histology (squamous-cell carcinoma) (%)	ECOG-PS 0–1 (%)	Stage IV (%)
Alessi et al. [9]	n.d	3; USA	234	68/73^a^	49	92%	13.2	83	100
Amrane et al. [10]	2017–2018	9; France	108	67	65	89%	25.9	65	87
Baldessari et al. [11]	2017–2018	1; Italy	44	70	59	98%	22.7	71	84
Banna et al. [12]	2016–2019	5; Europe	132	68	66	90%	27.0	84	86
Banna et al. [13]	2018–2019	n.d	128	70	66	100%	21.0	0	100
Banna et al. [14]	2020–2020	2; Europe	66	68	64	65%	42.0	91	89
Bureau et al. [15]—BTS ≤ 86 mm cohort^b^	2016–2020	3; France	50	63	56	94%	28.0	80	78
Bureau et al. [15]—BTS > 86 mm cohort^b^	2016–2020	3; France	46	63	80	98%	12.0	76	74
Cavaille et al. [16]	2017–2018	1; France	41	64	49	n.d	12.2	73	95
Chen et al. [17]	2017–2020	1; Shanghai	91	67	88	79%	46.0	100	65
Cortellini et al. [18]	2017–2019	34; Italy	1026	70	66	90%	24.2	83	100
Cramer-van der Welle et al. [19]	2015–2018	6; Netherlands	83	66	54	n.d	13.0	96	100
Dall’Olio et al. [20]	2017–2020	1; Italy	34	67^	65	n.d	15.0	77	100
Dudnik et al. [21]	2016–2020	4; Israel	203	68^	68	91%	16.8	68	96
Facchinetti et al. [22]	2017–2018	21; Italy	153	70	71	81%	16.0	0	94
Friedlaender et al. [23]	2016–2020	16; Europe	302	69	65	90%	25.0	100	100
Frost et al. [24]	2017–2018	4; Germany	119	68	58	92%	10.1	77	84
Frost et al. [25]	2017–2017	6; Germany	153	69	59	93%	21.0	75	81
Galan et al. [26]	2017–2020	1; Spain	88	67	78	91%	20.0	64	98
Geiger-Gritsch et al. [27]	2017–2018	6; Austria	42	68	62	95%	19.0	91	86
Grosjean et al. [28]—< 70 y cohort	2010–2019	2; USA	158	n.d	44	92%	14.0	73	68
Grosjean et al. [28]—≥ 70 y cohort	2010–2020	2; USA	169	n.d	51	93%	24.0	74	60
Hasegawa et al. [29]	2017–2019	2; Japan	51	70	78	86%	27.0	90	86
Holzman et al. [30]	2016–2020	5; Israel	302	70	66	89%	19.1	66	94
Hosoya et al. [31]	2017–2019	11; Japan	88	69^c^	84	89%	27.0	85	76
Ikezawa et al. [32]	2018–2020	34; Japan	166	74	79	85%	28.0	71	84
Imai et al. [33]	2017–2019	6; Japan	142	70	82	92%	30.1	78	74
Isono et al. [34]	2016–2020	1; Japan	38	72	74	87%	21.1	84	82
Ivanovic et al. [35]	2015–2018	1; Slovenia	26	66	62	77%	12.0	89	100
Kawachi et al. [36]	2017–2018	11; Japan	213	71	83	91%	26.0	81	68
Matsumoto et al. [37]	2016–2019	5; Japan	47	71	79	83%	31.3	100	66
Metro et al. [38]	2016–2019	15; Europe	282	69	64	91%	23.4	82	100
Metro et al. [39]	2016–2019	15; Europe	9	74	44	78%	22.2	89	100
Mountzios et al. [40]	2017–2019	14; Europe	265	67	66	90%	24.9	82	85
Mouritzen et al. [41]	2013–2018	Denmark	579	70	42	92%	23.0	85	81
Noordhof et al. [42]	2017–2020	Netherlands	595	65	50	n.d	0.0	77	100
Passaro et al. [43]	2017–2019	5; Italy	336	69	68	85%	18.5	87	100
Sanchez-Gastaldo et al. [44]	2017–2019	1; Spain	51	66	73	71%	37.3	76	100
Schakenraad et al. [45]	2015–2020	1; Netherlands	52	69	50	n.d	19.2	89	92
Takumida et al. [46]	2017–2020	1; Japan	89	69	73	85%	19.8	73	62
Tambo et al. [47]	2017–2018	7; Japan	95	72	75	81%	32.6	78	70
Velcheti et al. [48]—EHR cohort^d^	2016–2017	USA	423	72	54	92%	23.4	100	100
Velcheti et al. [48]—spotlight cohort^d^	2016–2017	USA	188	72	48	91%	24.5	100	85
Wakuda et al. [49]—BM cohort^e^	2017–2019	1; Japan	23	70	74	91%	17.0	91	83
Wakuda et al. [49]—non-BM cohort^e^	2017–2019	1; Japan	64	70	73	88%	19.0	90	55
Yamaguchi et al. [50]	2017–2019	1; Japan	72	70	85	89%	18.1	66	76

Note the table includes studies, in which IV stadium of the disease had at least 50% of patients

ECOG–PS, *Eastern Cooperative Oncology Group Performance Status*; stage, tumor stage according to TNM classification, range from I to IV; n.d. , no data (variable not reported in the source publication)

^a^ECOG-PS 0–2/ECOG-PS 2

^b^BTS > 86 mm and BTS ≤ 86 mm cohorts—in Bureau et al. [15] study cohorts were distinguished due to *baseline tumour size (BTS)*—the data given are for the cohort with BTS > 86 mm and the cohort with BTS ≤ 86 mm separately

^^c^Mean

^d^EHR and spotlight cohorts—in Velcheti et al. [48] two patient cohorts were included—one of patient data collected with *electronic health records* (EHR), and the second one containing data from the traditional Spotlight database

^e^BM cohort—in Wakuda et al. [49] study the cohort of patients with *brain metastases* (BM) and the cohort of patients with *no brain metastases* (non-BM) were included

**Table 2:** Pooled patients characteristics included in the analysis

Number of patients	7666
Age (years) [median (range)]	69 (63; 74)
Male [median (range)]	65.7% (42.0%; 87.9%)
Current or ex-smokers [median (range)]	90.0% (70.6%; 100%)
Squamous-cell carcinoma histology [median (range)]	21.7% (0.0%; 46.0%)
ECOG-PS 0–1 [median (range)]	81.3% (0%; 100%)
• 100% ECOG-PS 0–1 n (%)	5 (11%)
• 90–99% ECOG-PS 0–1 n (%)	6 (13%)
• 80–89% ECOG-PS 0–1 n (%)	14 (30%)
• 70–79% ECOG-PS 0–1 n (%)	14 (30%)
• 60–69% ECOG-PS 0–1 n (%)	5 (11%)
• 50–59% ECOG-PS 0–1 n (%)	0 (0%)
• < 50% ECOG-PS 0–1 n (%)	2 (4%)
Stage IV [median (range)]	85.8% (54%; 100%)
• 100% STAGE IV n (%)	13 (28%)
• 90–99% STAGE IV n (%)	6 (13%)
• 80–89% STAGE IV n (%)	14 (30%)
• 70–79% STAGE IV n (%)	5 (11%)
• 60–69% STAGE IV n (%)	7 (15%)
• 50–59% STAGE IV n (%)	1 (2%)
Brain metastases [median (range)]	19.3% (0.0%; 100.0%)
Previous local therapy of brain metastases^a^	68.8% (34.4%; 100,0%)
Liver metastases [median (range)]	13.0% (6.8%; 36.3%)

^a^Surgery or radiotherapy—data presented based on assumption that only patients with diagnosed brain metastases received treatment

**Fig. 1:**
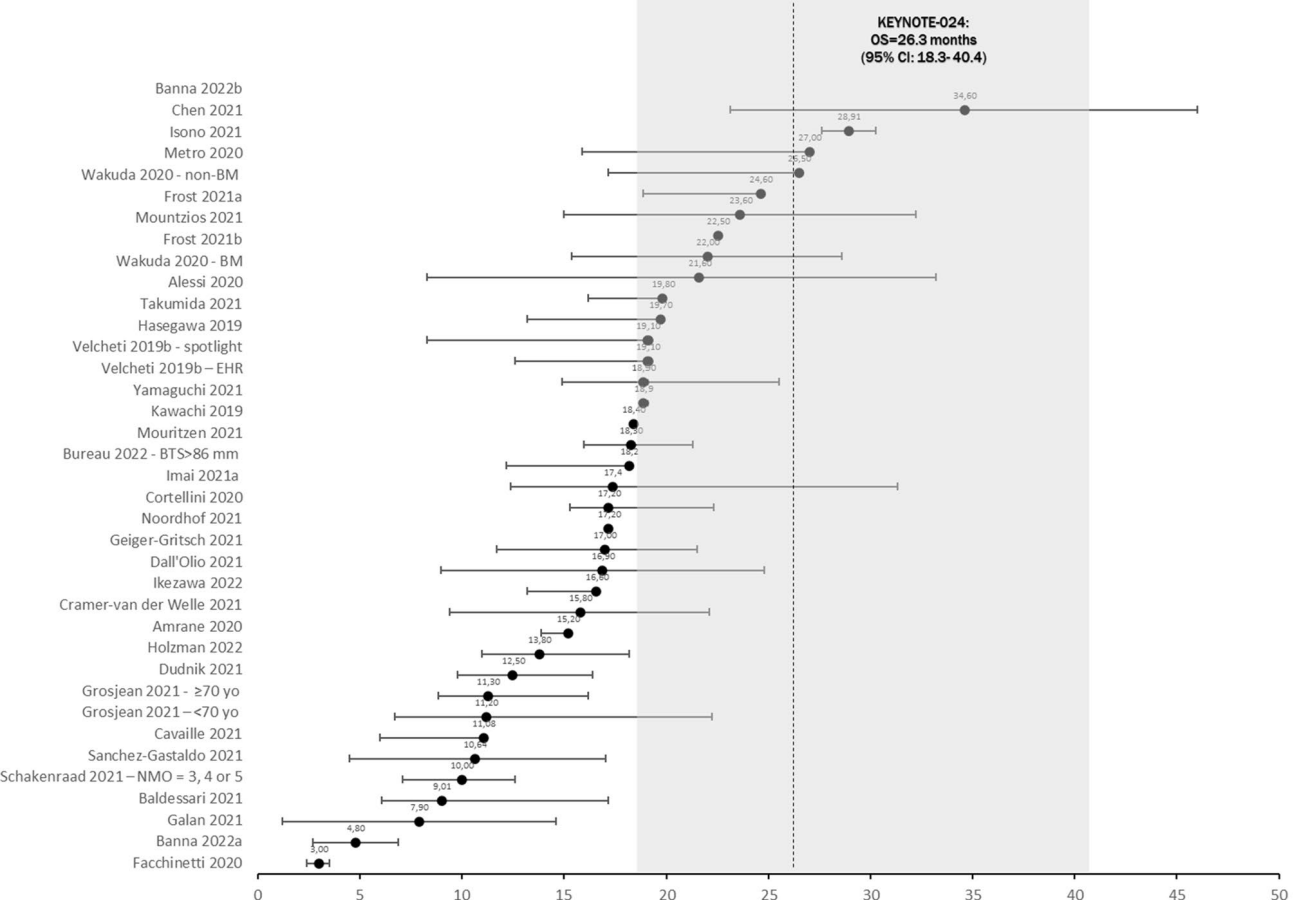
Median survival in RWE studies (all included trials)

Median survival times varied across studies with 3.0 months minimum and 34.6 months maximum. Most RWE studies reported median OS shorter to that reported in KEYNOTE-024; however, about half of the reported median OS were within the 95% confidence interval as reported in KN-024 trial (18.3–40.4 months; Fig. 1). Expectedly, moderately strong negative correlation was seen between percentage of ECOG-PS2+ patients and OS (Spearman correlation coefficient r_s_ = − 0.62).

**Fig. 2:**
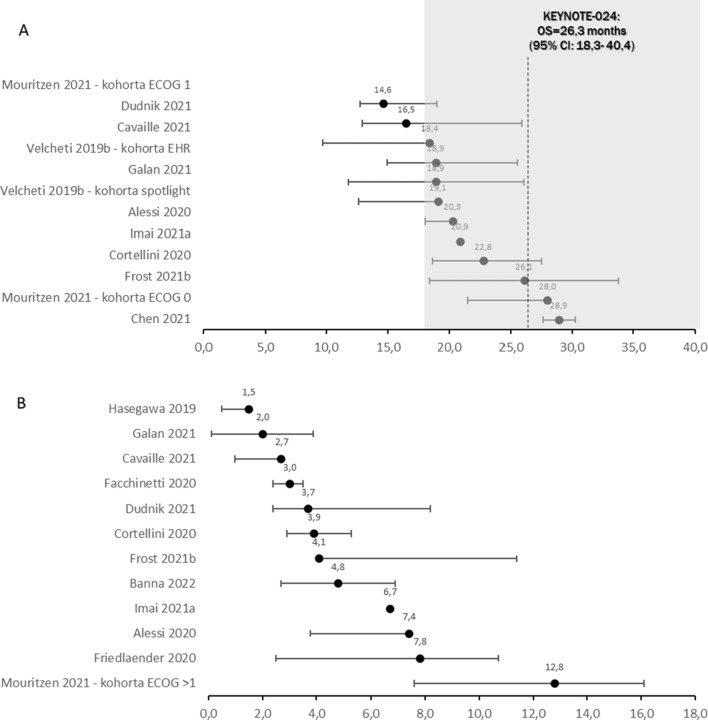
Median survival in ECOG-PS 0–1 (**A**) and ECOG-PS 2+ (**B**) patients

**Fig. 3:**
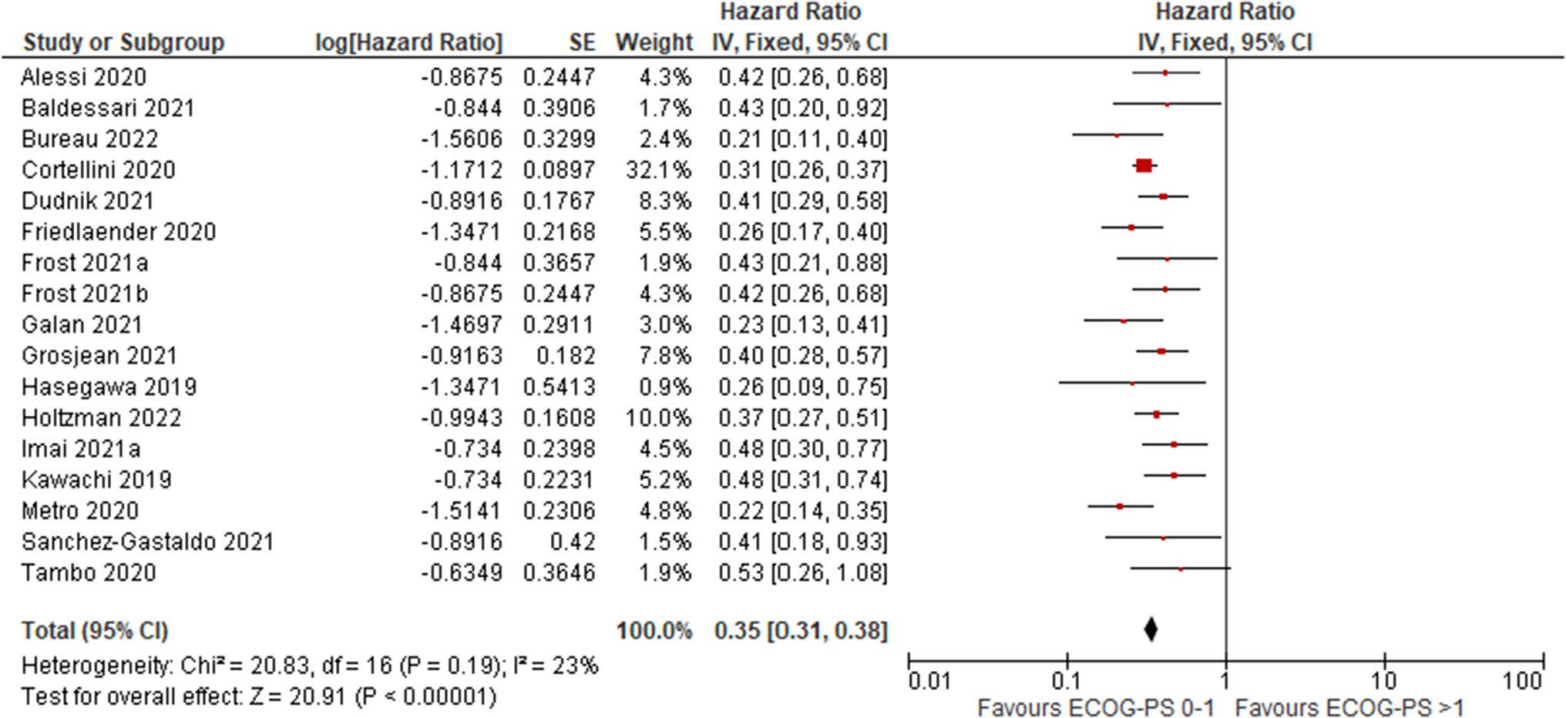
Pooled overall survival HR in ECOG-PS 0–1 vs ECOG-PS 2+ patients

When comparing OS for subpopulation with ECOG-PS 0–1 to subpopulation with ECOG-PS 2+ significant difference in favour of patients with better performance status was shown (median range: 14.3–28.9 months vs 1.5–12.8 months)—see Fig. 2A and B. Pooled HR for OS data showed significant difference in favour of ECOG-PS 0–1 population (HR = 0.35; 95%CI: 0.31, 0.38; p < 0.001)—see Fig. 3. In 3 studies of population analogous to KEYNOTE-024 (i.e. only patients at stage IV and ECOG-PS 0–1) median OS months were 22.8, 20.3 and 18.9 [9, 18, 48—EHR cohort, respectively]. As presented on Fig. 2A most results in ECOG-PS 0–1 population were within 95% confidence interval for OS reported in KEYNOTE-024 trial, while in ECOG-PS 2+ population none of OS reached 95% CI range from the clinical trial (Fig. 2B).

The 1-year and 2-years OS rates were achieved in 57.0% (median; range: 21.4%–92.0%) and 42.5% (median; range: 8.0%–79.0%)—see Table 3. Those values are lower to those reported in KEYNOTE-024 trial (70.3% and 54.8%, respectively).

**Table 3:** Annual and 2-years overall survival rates

Study	Stage IV (%)	1 year survival			2 year survival		
		N	%	95% CI	N	%	95% CI
Banna et al. [13, 14]	100	128	21.4%	20.5–22.5%	128	16.3%	15.6–17.2%
Cramer-van der Welle et al. [19]	100	83	57.0%	n.d	n.d	n.d	n.d
Frost et al. [24]—KRAS^other^/TP53^mut^ cohort	100	14	48.0%	n.d	14	30.0%	n.d
Ivanovic et al. [35]	100	26	62.0%	45.0–83.0%	n.d	n.d	n.d
Metro et al. [39]	100	9	55.5%	b.d	n.d	n.d	n.d
Noordhof et al. [42]	100	595	57.0%	53.0–61.0%	595	44.0%	39.0–48.0%
Passaro et al. [43]	100	n.d	n.d	n.d	336	53.3%	n.d
Velcheti et al. [48]—EHR cohort^a^	100	423	59.1%	54.0–63.9%	n.d	n.d	n.d
Alessi et al. [9]—ECOG-PS 0–1 cohort^b^	100*	195	73.0%	66.7–79.8%	n.d	n.d	n.d
Alessi et al. [9]—ECOG-PS ≥ 2 cohort^b^	100*	39	41.0%	27.2–60.8%	n.d	n.d	n.d
Schakenraad et al. [45]—NMO = 3, 4 or 5 cohort^c^	100	14	42.0%^d^	n.d	14	24.0%^d^	n.d
Galan et al. [26]	98	88	36.0%	n.d	n.d	n.d	n.d
Cavaille et al. [16]	95	41	85.0%	75.0–97.0%	n.d	n.d	n.d
Facchinetti et al. [22]	94	153	29.0%	n.d	153	8.0%	n.d
Banna et al. [12]	86	n.d	n.d	n.d	128	52.0%	49.3–55.0%
Frost et al. [24]—KRAS^G12C^/TP53^wt^ cohort^e^	84*	n.d	79.0%	n.d	n.d	41.0%	n.d
Frost et al. [24]—KRAS^other^/TP53^wt^ cohort^e^	84*	n.d	81.0%	n.d	n.d	44.0%	n.d
Frost et al. [24]—KRAS^G12C^/TP53^mut^ cohort^e^	83	12	92.0%	n.d	12	79.0%	n.d
Velcheti et al. [48]—spotlight cohort^b^	85	188	60.4%	52.7–67.2%	n.d	n.d	n.d
Baldessari et al. [11]	84	44	45.0%	33.0–60.0%	44	40.0%	24.0–54.0%
Bureau et al. [15]—BTS > 86 mm cohort^f^	78	n.d	n.d	n.d	46	40.1%	27.9–57.6%
Bureau et al. [15]—BTS ≤ 86 mm cohort^f^	74	n.d	n.d	n.d	50	69.8%	56.9–85.6%
Chen et al. [17]	65	91	76.1%	n.d	n.d	n.d	n.d

OS, overall survival; N, number of all patients in the group; 95% CI, 95% confidence interval; n.d., no data (variable not reported in the source publication)

*Entire study population data (no specific subgroups data available)

^a^EHR cohort data—Velcheti et al. [48] included two patient cohorts—one with patient data obtained from electronic health records (EHR) and the other with data from the traditional Spotlight database, this cohort covers data from electronic databases

^b^In the Alessi et al. [9] study, the results were divided into subpopulations according to the degree of patient performance determined in accordance with the ECOG-PS questionnaire—the group with a value of 0–1 and a group with a value above 1 were distinguished

^c^Data in subpopulations by number of metastatic organs—this subpopulation is for patients with 3, 4 or 5 metastatic organs

^d^Data read from the graph using the WebPlotDigitizer software

^e^In Frost et al. [24] results for OS in the form of a binary value (percentage of patients with OS) are presented in subpopulations according to the presence and type of KRAS/TP53 mutation

^f^Cohort BTS > 86 mm and BTS ≤ 86 mm—in the Bureau et al. [15] study, cohorts were distinguished according to the baseline tumor size—the data provided for the cohort with BTS > 86 mm and the cohort with BTS < 86 mm separately

**Fig. 4:**
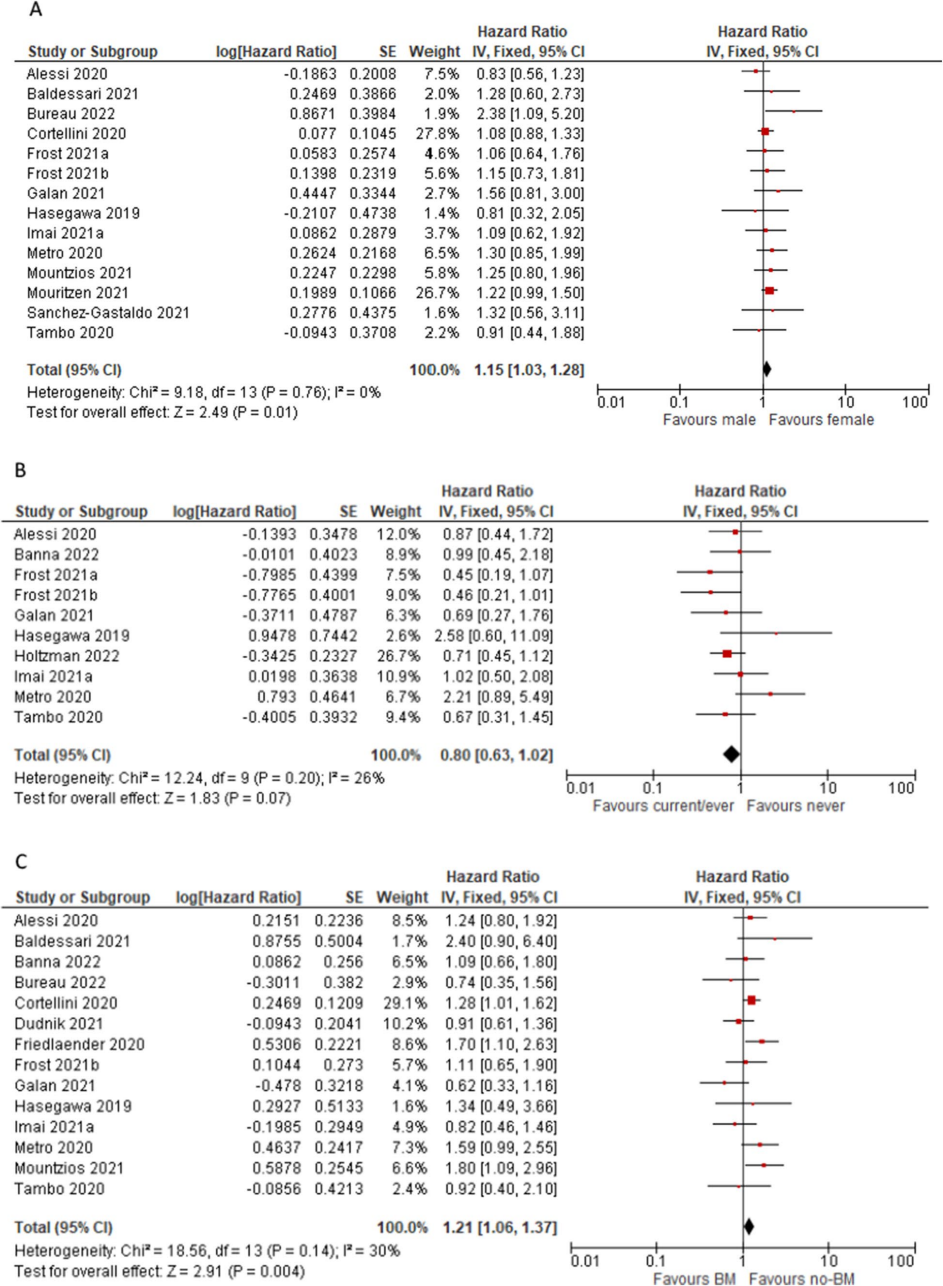

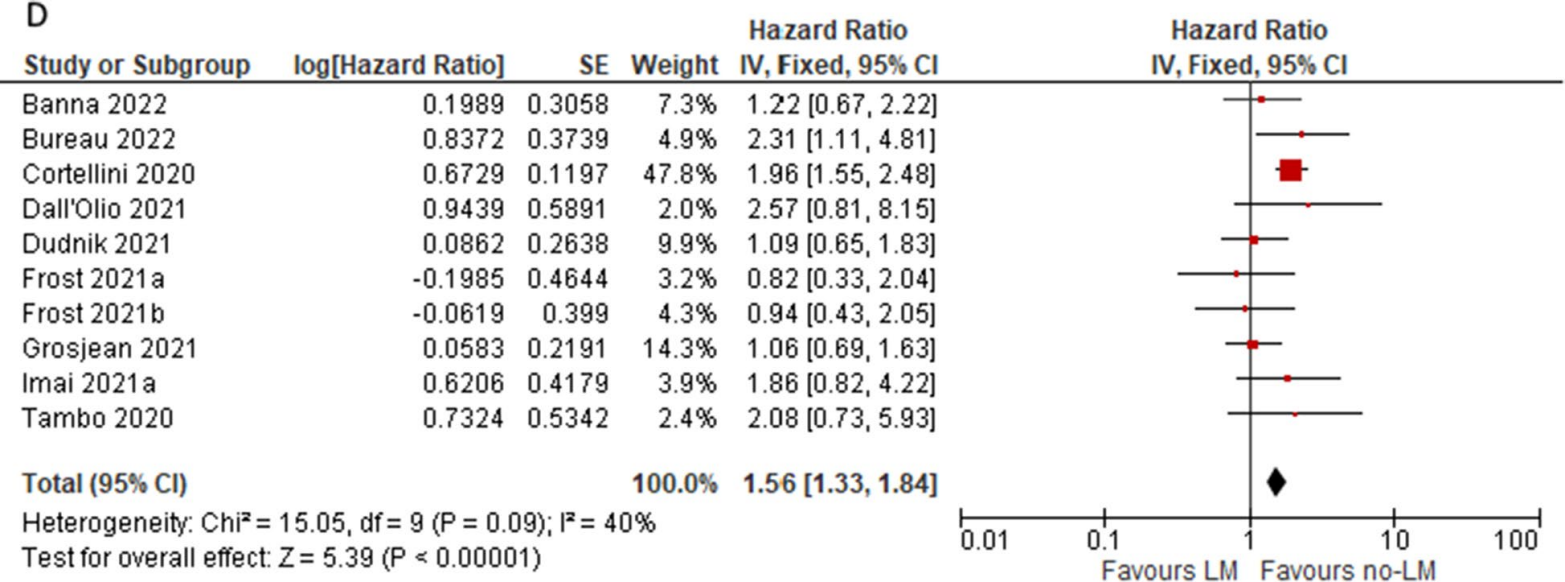
Pooled overall survival HR in patients subgroups: males vs females (**A**), never smokers vs current/ever smokers (**B**) patients with and without brain metastases (**C**) and patients with and without liver metastases (**D**)

Pooled HR for OS data showed significant difference in favour of females (HR = 1.15; 95%CI 1.03, 1.28; p = 0.01) and suggested a trend toward less benefit in never smokers; patients without brain metastases (HR = 1.21; 95%CI 1.06, 1.21; p = 0.004) as well as with no evidence of liver metastases (HR = 1.56; 95%CI 1.33, 1.84; p < 0.001) have significantly better prognosis. See Fig. 4A–D, respectively.

## Discussion

We identified substantial number of RWE studies covering over 7600 previously untreated patients with metastatic NSCLC receiving pembrolizumab monotherapy. In general, the OS results vary across all analysed studies and we believe the differences are due to high heterogeneity of population included in each study in terms of known prognostic factors (i.e. ECOG-PS, tumor stage) as well as local practice on supportive management. Approximately half of reported OS medians were in range of 95% CI OS data form KEYNOTE-024 trial. Those observations are not surprising as KEYNOTE-024 trial covered only ECOG-PS 0–1 patients, and we have shown that ECOG-PS is an important factor affecting outcome. Similar observation was reported previously for pembrolizumab in pre-treated patients with NSCLC [51]. Also studies for other immune checkpoint inhibitors used for advanced NSCLC showed that patients with impaired performance status had significantly shorter survival compared to those with better performance status [52].

It’s worth to mention that KEYNOTE-024 trial covered only stage IV patients, while in RWE studies patients at stage III were also included. However percentage of patients with brain metastasis were overrepresented in real world data (19.6%) compared to KEYNOTE-024 trial (11.7% in pembrolizumab arm), and contrary to KEYNOTE-024 trial in real world settings not all patients received local treatment for brain metastasis. As expected, pooled HR data showed patients with brain or liver metastasis are at higher risk of death compared to patients without brain metastasis.

Review of RWE literature has shown that in real practice pembrolizumab monotherapy in patients with high PD-L1 expression may produce almost the same survival results as reported in the KEYNOTE-024 trial provided they have comparable stage and performance status. Although ESMO guidelines claim systemic therapy should be offered to all stage IV patients with PS 0–2, RWE data showed substantially worse outcomes in patients with ECOG-PS ≥ 2 compared to ECOG-PS 0–1 patients. In fact, in some countries (i.e. Poland) the use of immunotherapy is limited only to patients with ECOG-PS 0–1, what is in line with KEYNOTE-024 trial inclusion criteria. However, PePS2 trial, the only prospective phase 2 study that evaluated pembrolizumab monotherapy in patients with NSCLC and ECOG-PS 2, reported median OS of 9.8 (95%CI 7.1–14.6) months—values higher to those observed in the majority of ECOG-PS 2 RWE studies included in this review (1.5–12.8 months) [53]. On the other hand, RWE showed patients without distant metastases may also benefit from pembrolizumab monotherapy, but this findings has to be verify in controlled clinical study. Although a meta-analysis of clinical trials showed that pembrolizumab significantly improved overall survival in male individuals regardless of treatment line and regimen, we observed better outcomes in females [54].

Our secondary analysis has limitations, which result mainly from non-randomized settings and corresponding selection bias and many confounding variables we were unable to control for, even though the study focused on advanced NSCLC patients naïve to systemic treatment with similar characteristics of stage and performance status to those of KEYNOTE-024 trial. Many variables reflecting not only different patients’ characteristics, but also clinical practice arrangements, contribute to heterogeneity and affect the generalizability of the study results. Moreover, most likely, everyday clinical practice and locally arranged patients' access to treatment not harmonized with predefined study protocol most likely could substantially affect generalizability. We focused on mortality to restrain missing data and measurement bias, but anticipated high heterogeneity, which could not be further limited with no access to individual patients data but only aggregated statistics reported in individual studies. For the same reason, our analysis focused mainly on median overall survival as most consistently reported; the only pooled statistic we could reliably provide through meta-analysis, with low heterogeneity, was the overall survival hazard ratio (HR). Certainly, general treatment efficacy claims are unjustified and were not intended as the study explored real-world outcomes in uncontrolled and pragmatic settings.

We believe more RWE in the population close to that of the KEYNOTE-024 trial and specific subgroups should be collected to credibly explore pembrolizumab efficacy in real-world practice. It should not restrain from well-designed controlled trials to confirm pembrolizumab efficacy in new specific populations or clinical settings.

In conclusion, RWE studies of over 7600 advanced NSCLC patients new to systemic treatment displayed considerable variability in survival outcomes. While most studies reported a median OS shorter than that seen in the KEYNOTE-024 trial, patients with similar stage and performance status benefited equally from pembrolizumab monotherapy, with survival outcomes consistent with the clinical trial findings.

### Supplementary Information

Supplementary Material 1.

Supplementary Material 2.

Supplementary Material 3.

## Data Availability

Publicly available data.
