# Supplementary figures and images for: Pembrolizumab monotherapy survival benefits in metastatic non-small-cell lung cancer: a systematic review of real-world data
Source: Discov Oncol. 2024 Jul 24;15:303. doi: 10.1007/s12672-024-01153-3 (PMC11269554; doi:10.1007/s12672-024-01153-3)

# Supplementary materials

Figure 1. PRISMA diagram.


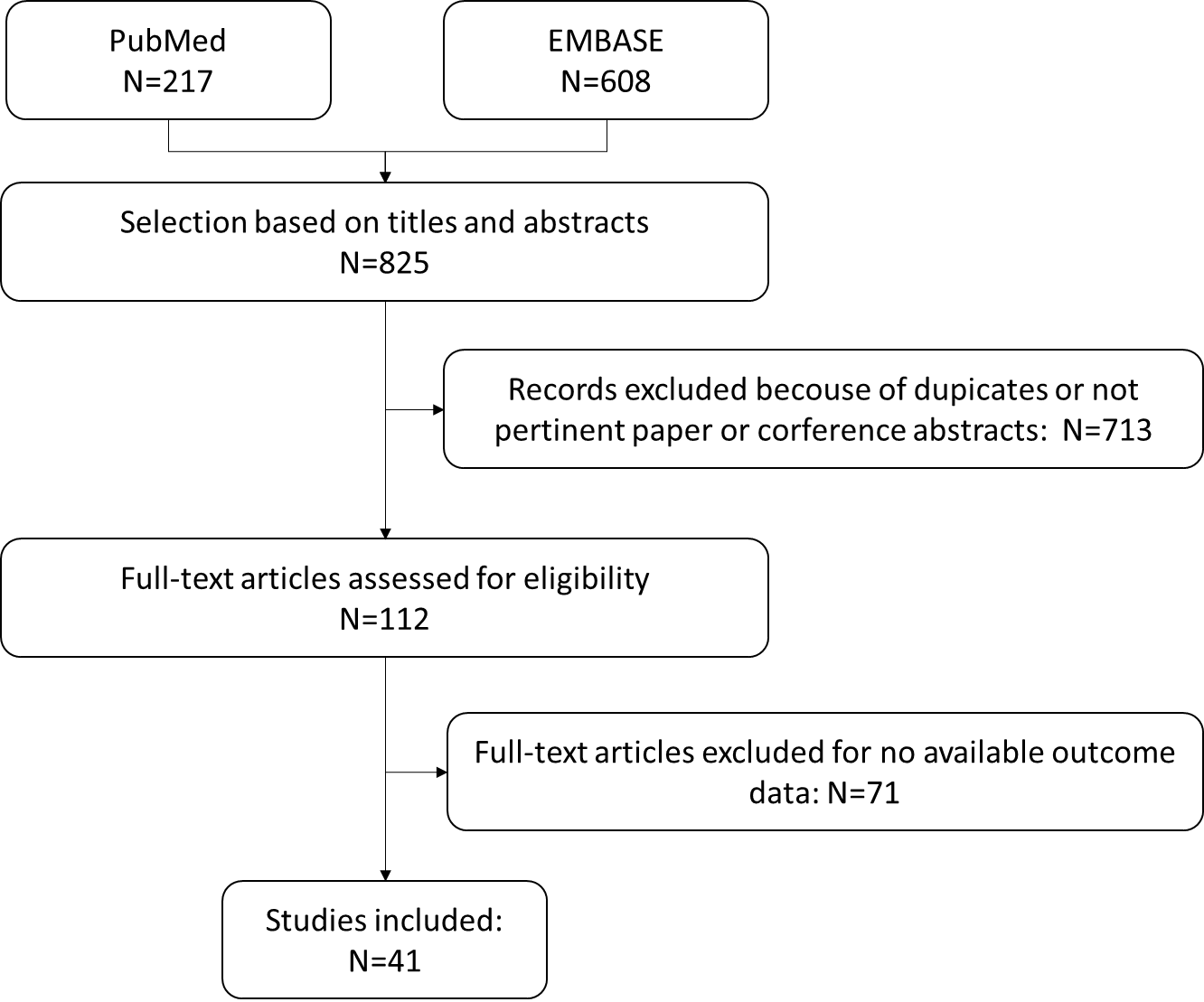

Supplement: Supplementary file 1 — Supplementary Material 1. [file 12672_2024_1153_MOESM1_ESM.docx]
