# Supplementary material for: Pembrolizumab monotherapy survival benefits in metastatic non-small-cell lung cancer: a systematic review of real-world data
Source: Discov Oncol. 2024 Jul 24;15:303. doi: 10.1007/s12672-024-01153-3 (PMC11269554; doi:10.1007/s12672-024-01153-3)
Supplement: Supplementary file 2 — Supplementary Material 2. [file 12672_2024_1153_MOESM2_ESM.docx]

# Supplementary materials

Table. 1. Search strategy in PubMed (MEDLINE) – 17th June 2022.

| Search number | Query | Results |
| --- | --- | --- |
| #1 | Carcinoma*, Non Small Cell Lung[MeSH Terms] | 2,626 |
| #2 | Carcinoma*, Non Small Cell Lung[Text Word] | 71,577 |
| #3 | Non-Small-Cell Lung Carcinoma*[Text Word] | 6,059 |
| #4 | Carcinoma, Non-Small Cell Lung[Text Word] | 65,031 |
| #5 | Non-Small Cell Lung Cancer[Text Word] | 69,708 |
| #6 | Nonsmall Cell Lung Cancer[Text Word] | 2,687 |
| #7 | Lung Neoplasm*[Text Word] | 246,036 |
| #8 | Adenocarcinoma[Text Word] | 256,799 |
| #9 | Carcinoma, Squamous Cell[Text Word] | 137,536 |
| #10 | Large Cell Carcinoma*[Text Word] | 2,291 |
| #11 | #1 OR #2 OR #3 OR #4 OR #5 OR #6 OR #7 OR #8 OR #9 OR #10 | 584,789 |
| #12 | pembrolizumab[Text Word] | 7,307 |
| #13 | SCH-900475[Text Word] | 2 |
| #14 | Lambrolizumab[Text Word] | 23 |
| #15 | MK-3475[Text Word] | 54 |
| #16 | Keytruda[Text Word] | 104 |
| #17 | #12 OR #13 OR #14 OR #15 OR #16 | 7,332 |
| #18 | #11 AND #17 | 2,395 |
| #19 | observational stud* | 280,461 |
| #20 | Retrospective | 1,300,923 |
| #21 | real world | 78,17 |
| #22 | case control | 1,607,001 |
| #23 | cohort stud* | 2,627,973 |
| #24 | Longitudinal | 374,543 |
| #25 | cross sectional | 592,868 |
| #26 | #19 OR #20 OR #21 OR #22 OR #23 OR #24 OR #25 | 4,038,169 |
| #27 | #18 AND #26 | 641 |
| #28 | first line | 256,141 |
| #29 | #27 AND #28 | 217 |
