# Supplementary material for: Pembrolizumab monotherapy survival benefits in metastatic non-small-cell lung cancer: a systematic review of real-world data
Source: Discov Oncol. 2024 Jul 24;15:303. doi: 10.1007/s12672-024-01153-3 (PMC11269554; doi:10.1007/s12672-024-01153-3)
Supplement: Supplementary file 3 — Supplementary Material 3. [file 12672_2024_1153_MOESM3_ESM.docx]

# Supplementary materials

Table 2. Search strategy in EMBASE – 17th June 2022.

| Search number | Query | Results |
| --- | --- | --- |
| #1 | ‘Non small cel lung cancer’/exp OR ‘non small cel lung cancer’ | 198,334 |
| #2 | ‘pembrolizumab’/exp OR ‘pembrolizumab’ | 28,511 |
| #3 | ‘first-line’ | 170,142 |
| #4 | #1 AND #2 AND #3 | 1,559 |
| #5 | #4 AND (‘clinical article’/de OR ‘observational study’/de OD retrospective study’/de) | 608 |
